# Nonalcoholic Fatty Liver Disease Aggravated the Severity of Acute Pancreatitis in Patients

**DOI:** 10.1155/2019/9583790

**Published:** 2019-01-22

**Authors:** Dacheng Wu, Min Zhang, Songxin Xu, Keyan Wu, Ningzhi Wang, Yuanzhi Wang, Jian Wu, Guotao Lu, Weijuan Gong, Yanbing Ding, Weiming Xiao

**Affiliations:** ^1^Department of Gastroenterology, Affiliated Hospital of Yangzhou University, Yangzhou University, No. 386 Hanjiang Media Road, Yangzhou 225000, Jiangsu, China; ^2^Department of Immunology, School of Medicine, Yangzhou University, Yangzhou, China

## Abstract

**Background and Aim:**

The incidence of nonalcoholic fatty liver disease (NAFLD) as a metabolic disease is increasing annually. In the present study, we aimed to explore the influence of NAFLD on the severity of acute pancreatitis (AP).

**Methods:**

The severity of AP was diagnosed and analyzed according to the 2012 revised Atlanta Classification. Outcome variables, including the severity of AP, organ failure (all types of organ failure), and systemic inflammatory response syndrome (SIRS), were compared for patients with and without NAFLD.

**Results:**

Six hundred and fifty-six patients were enrolled in the study and were divided into two groups according to the presence or absence of NAFLD. The non-NAFLD group contained 278 patients and the main etiology in this group was gallstone. The NAFLD group consisted of 378 patients and the main etiology was hyperlipidemia. The incidence of mild AP, moderately severe AP, and severe AP was 77.30%, 18.3%, and 4.3% in the non-NAFLD group and 58.2%, 33.9%, and 7.9% in the NAFLD group, respectively. There were significant differences between the two groups according to the severity of AP (P ≤ 0.001). In addition, the Ranson and BISAP scores as well as the incidence of SIRS and organ failure in the NAFLD group were higher than those in the non-NAFLD group (all P < 0.05). The patients were further divided into non-NAFLD, mild-NAFLD, and moderate-severe NAFLD (M+S-NAFLD) groups. The results showed that the severity of AP increased gradually from the non-NAFLD group to the M+S-NAFLD group. In addition, the incidence rates of SIRS and organ failure showed an upward trend with the aggravation of fatty liver severity. Multivariate logistic analysis showed that patients with NAFLD, especially those with M+S-NAFLD, had higher risks of SIRS and organ failure.

**Conclusions:**

Compared with non-NAFLD, NAFLD has a clinically relevant impact on the severity of AP and may be an early prognostic parameter for patients with AP.

## 1. Introduction

Acute pancreatitis (AP) is an inflammatory disease of the pancreas, with 10–20% of patients progressing to multiple organ failure coupled with a high mortality rate. The incidence of AP is increasing year by year, consistent with an increase in the number of people with metabolic syndrome. The incidence of local and systemic complications, especially mortality in patients with AP with metabolic syndrome, is noteworthy [1]. Metabolic syndrome is a clinical diagnosis based on the identification of related metabolic status. It can increase the risk of cardiovascular diseases, including diabetes, dyslipidemia, arterial hypertension, and abdominal obesity [2]. Abdominal obesity, a typical phenotype of metabolic syndrome, has been demonstrated to be an independent risk factor for AP [3]. Many clinical studies have confirmed that abdominal obesity can increase the severity of AP, prolong hospital stay, and increase the intensive care unit occupancy rate and mortality [4, 5].

Nonalcoholic fatty liver disease (NAFLD) is a phenotype of metabolic syndrome in the liver. NAFLD is related to all the components of metabolic syndrome and may be considered an additional component of the disease itself [6]. NAFLD is characterized by excessive hepatic fat accumulation, associated with insulin resistance (IR), and defined by the presence of steatosis in >5% of hepatocytes according to histological analysis or by a proton density fat fraction (providing a rough estimation of the volume fraction of fatty material in the liver)>5.6% assessed by proton magnetic resonance spectroscopy (^1^H-MRS) or quantitative fat/water selective magnetic resonance imaging (MRI) [7]. The incidence of NAFLD worldwide is approximately 28.01–52.34/1,000 [8], and NAFLD is increasingly recognized in the West. NAFLD is one of the main causes of chronic liver disease, which has become one of the major causes of liver disease-related morbidity and mortality in Western countries [9]. An independent epidemiological survey showed that, from 2007 to 2013, the prevalence of NAFLD in the general population increased from 23.5% to 44.3% among men and from 17.6% to 43.1% among women [10]. The prevalence of NAFLD in the average adult rose from 15% to more than 31% over a 10-year period, according to a survey in Shanghai and Beijing, China [11].

Studies have been conducted on the association between fatty liver and AP [12, 13]. Xu et al. separated 2,671 patients with pancreatitis into a fatty liver group and a non-NAFLD group. The results of the study showed that fatty liver can increase the severity of AP. However, the association between NAFLD and the severity and clinical outcomes of AP has been poorly studied. Therefore, we aimed to investigate the effect of NAFLD as a manifestation of metabolic disease on the severity of AP.

## 2. Methods

### 2.1. Inclusion and Exclusion Criteria

A retrospective analysis of 1186 patients with AP was conducted from January 2012 to December 2016. The diagnostic criteria for AP included three items: (1) typical clinical symptoms with persistent abdominal pain; (2) serum amylase and/or lipase levels three times higher than the normal upper limit; and (3) characteristic results of abdominal imaging [14]. Patients suffering from cirrhosis, hepatocellular carcinoma, alcoholic fatty liver, or chronic pancreatitis as well as those who had undergone splenectomy, were pregnant, were younger than 18 or older than 60 years, had been hospitalized repeatedly, or had incomplete medical data were excluded from the analysis. The cause of AP was considered to be biliary if gallstones or biliary sludge was observed on imaging examinations, including computed tomography (CT), magnetic resonance cholangiopancreatography, and ultrasonography [15, 16]. Hypertriglyceridemic acute pancreatitis (HTG-AP) was characterized by the presence of serum hypertriglyceridemia (≥1000 mg/dL) or by visible lactescent blood with serum hypertriglyceridemia 500–1000mg/dL without any other causes [17–19]. The exclusion criteria were age >70 or <18 years, recurrent pancreatitis, malignant tumor, ascites, pregnancy, and incomplete information. Due to the retrospective characteristics of the study from 2012 to 2016, informed consent was waived and the study was approved by the Ethics Committees of our hospital.

### 2.2. Diagnostic Criteria for NAFLD

Abdominal computed tomography (CT) was used to determine the presence of fatty liver based on CT values for the liver and spleen. Patients with a history of alcoholic consumption (history of drinking or equivalent alcohol consumption of more than 140 g/week for men and more than 70 g/week for women), viral hepatitis, drug-induced hepatitis, total parenteral nutrition, hepatolenticular degeneration, autoimmune liver disease, and other specific diseases that can lead to fatty liver were excluded [20].

### 2.3. NAFLD Classification Criteria

According to the literature, NAFLD is diagnosed based on the ratio of the CT values for the liver and spleen, which are measured in the range of 88–92 mm. Mild-NAFLD is defined as a liver/spleen CT ratio less than or equal to 1. If the ratio is higher than 0.5 and lower than or equal to 0.7, the disease is classified as moderate NAFLD. Severe NAFLD is defined as a ratio lower than or equal to 0.5 [21].

### 2.4. Severity Assessment of AP

According to the revised Atlanta Classification, AP severity is divided into three groups: mild, moderately severe, and severe. Mild AP (MAP) involves no organ failure and no local or systemic complications. Moderately severe AP (MSAP) is characterized by temporary organ failure and/or local or systemic complications within 48 hours without persistent organ failure. Severe AP (SAP) is defined as persistent organ failure lasting more than 48 hours [22].

### 2.5. Criteria for Systemic Inflammatory Response Syndrome (SIRS)

SIRS is defined as the existence of two or more of the following: (1) temperature > 38°C or < 36°C; (2) heart rate > 90 beats/min or hypotension (systolic blood pressure <90 mmHg, or >40 mmHg lower than the baseline); (3) shortness of breath (>20 beats/min) or hyperventilation (PaCO2 <32 mmHg); and (4) peripheral blood leukocyte count >12 × 10^∧^9/L or neutral rod-shaped granulocyte ratio >10%. However, other factors that may cause the above acute abnormal changes should be excluded [23].

### 2.6. Data Analysis

Data were analyzed with SPSS 16.0. Continuous variables were represented as the mean ± standard deviation (SD) or the median (quartile spacing) and compared using the T test. Data were evaluated based on the quantity and proportion, and descriptive statistics were used to analyze the baseline characteristics of the population; the severity was assessed using one-way analysis of variance or the Pearson chi-square test. The Kruskal–Wallis test was used for contingency table analysis. To determine whether the severity of NAFLD was related to organ failure and SIRS, the Spearman test was used. Age, gender, Body Mass Index (BMI), hypertension, diabetes, coronary heart disease (CHD), smoking, and NAFLD were set as independent variables for multivariable regression analyses, and organ failure was set as a dependent variable. Comparing the characteristics and variables among the groups, P < 0.05 indicated significant differences.

## 3. Results

### 3.1. General Baseline Situation

**Figure 1:**
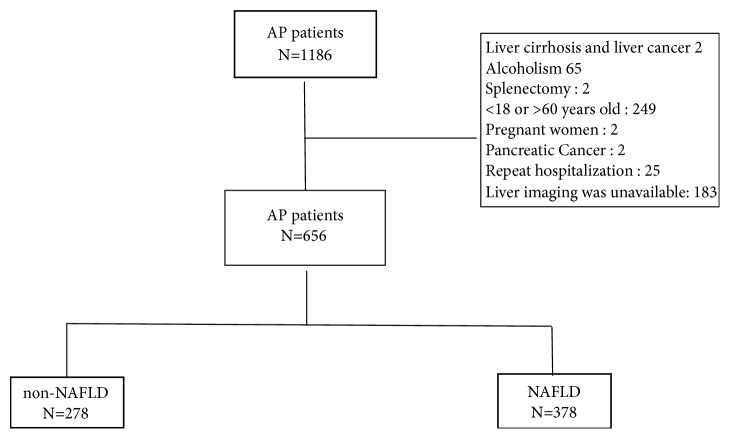
The distribution of AP patients.

A total of 1,186 patients with AP were diagnosed from January 2012 to December 2016. Five hundred and thirty were excluded according to the exclusion criteria, and 656 were enrolled in the study (as shown in the flowchart, Figure 1). Demographic characteristics are shown in Table 1. The average age of the patients was 43.93 ± 9.81 years and men accounted for 63.0%. The patient population comprised 20.4% of smokers, 7.8% of patients with hypertension, 15.9% of patients with diabetes mellitus, and 1.4% of patients with CHD. The patients were divided into non-NAFLD (278 patients) and NAFLD (378 patients) groups based on the CT results. The average age was 45.96 ± 10.20 years for the non-NAFLD group and 42.44 ± 9.24 years for the NAFLD group. The proportion of diabetes mellitus in the NAFLD group was also higher than that in the non-NAFLD group (19.0% versus 11.5%), but there was no significant difference in drinking.

The causes of AP include gallstone, hyperlipidemia, and others. As shown in Table 1, the percentage of gallstone, hyperlipidemia, and others was 31.6%, 48.1%, and 20.3% in the cohort, respectively. Interestingly, the main etiology in the non-NAFLD group was gallstone pancreatitis (49.0%), whereas that in the NAFLD group was hyperlipidemia pancreatitis (65.3%).

### 3.2. Comparison of the Influence of the Presence and Severity of NAFLD in Patients with AP

We compared the laboratory indexes in the two groups and found that the white blood cell (WBC) and triglyceride concentrations, which are used to determine the severity of AP, were significantly higher in the NAFLD group. However, the serum creatinine was not significantly different in the two groups. These results showed that the severity of AP in patients with NAFLD was significantly greater than that in patients without NAFLD. In addition, compared with the non-NAFLD group, the incidence of MAP was lower (77.3% versus 58.2%) and the incidence of SAP was higher (4.3% versus 7.9%) in the NAFLD group. There was a significant difference between the two groups in the severity of AP (P ≤ 0.001). Furthermore, the clinical scores and related complications of AP in the non-NAFLD and NAFLD groups were compared. The results showed that both the clinical scores (Ranson and BISAP scores) and related complications including SIRS and organ failure (all types of organ failure) were more serious in the NAFLD group (all P< 0.05 Table 2).

**Figure 2:**
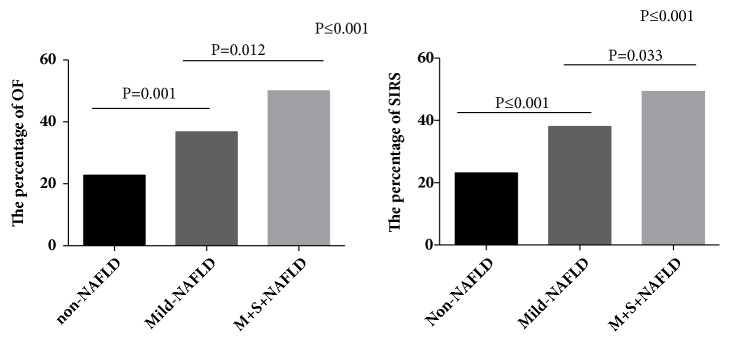
Comparison of SIRS and organ failure with non-NAFLD, mild-NAFLD, and M+S-NAFLD. OF: organ failure (all types organ failure); non-NAFLD: without NAFLD. Mild-NAFLD: mild nonalcoholic fatty liver disease; M+S-NAFLD: moderate-severe nonalcoholic fatty liver disease.

Based on the above results, the patients were further divided into non-NAFLD, mild-NAFLD, and moderate-severe NAFLD (M+S-NAFLD) groups according to the ratio of the CT value for the liver and spleen. As shown in Table 3, from the non-NAFLD group to the M+S-NAFLD group, the MAP ratio decreased, while the MSAP and SAP ratios increased (P ≤ 0.001). In addition, the results showed that both the incidence of SIRS and organ failure in the M+S-NAFLD group were scientifically higher than those in the non-NAFLD group, which was consistent with the above results. Furthermore, the incidence of SIRS and organ failure showed an upward trend with the aggravation of the severity of NAFLD (P trend <0.001; Figure 2).

### 3.3. Logistic Regression Analysis of Organ Failure in Patients with AP

Finally, we analyzed whether organ failure correlated with the epidemiology and clinical features of NAFLD. Multivariate logistic regression analysis was performed, and the results showed that patients with mild-NAFLD had a risk of organ failure 1.771 times greater than those without NAFLD (95% confidence interval [CI] = 1.080-2.903 and P = 0.023). Furthermore, patients with M+S-NAFLD had a 3.115 times greater risk of organ failure than those without NAFLD (95% CI = 1.766-5.493 and P ≤ 0.001). It is worth noting that patients with high TG may have a greater risk of organ failure (odds ratio = 1.026, 95% CI = 1.001-1.052, and P = 0.040 Table 4).

## 4. Discussion

Clinically, there are many causes of AP, including gallstone, alcohol, and hyperlipidemia. Gallstone is the primary cause globally, whereas in China, hyperlipidemia has exceeded alcohol to become the second major cause of pancreatitis [15, 19]. In the present study, patients with gallstone, hyperlipidemia pancreatitis (HTG-AP) and others accounted for 31.6%, 48.1%, and 20.3%, respectively. Further analysis of data revealed that the main cause of AP in the non-NAFLD group was gallstone, accounting for approximately 49.0%, whereas hyperlipidemia pancreatitis had the highest incidence in the NAFLD group (65.3%). The higher proportion of HTG-AP was probably because some biliary patients may have been excluded from the cohort without CT examination.

The results of this study showed that the severity of AP, including the clinical score, incidence of SIRS, and organ failure, in the NAFLD group was scientifically higher than that in the non-NAFLD group, which was consistent with the results reported by Xu and Mikolasevic [14, 24]. In addition, we found that the incidence of SIRS and organ failure showed an upward trend with the aggravation of the severity of NAFLD (P trend <0.001). All these findings imply that the severity of NAFLD has an impact on the course of AP. NAFLD is well known to be associated with other metabolic diseases, such as obesity, diabetes, and hyperlipidemia, and these metabolic diseases have a clear role in the severity of AP. In light of this, we further performed logistic regression analysis and determined that NAFLD was an independent risk factor for AP.

The mechanism by which NAFLD exacerbates pancreatitis remains to be elucidated. Patients with NAFLD are often associated with obesity. In our study, the BMI of NAFLD patients was 26.90 ± 3.45. The body is in a chronic inflammatory process for a long time in obesity patients, which makes the inflammatory factor response easy to expand. And NAFLD itself is an inflammatory disease that promotes chronic systemic inflammation [25–27], which may be an important reason for its exacerbation of AP. Secondly, in theory, Kupffer cells which are resident macrophages of the liver that represent approximately 70% of the liver's total macrophages play a very important role in the pathogenesis of AP by releasing a large number of inflammatory factors [28]. In the condition of NAFLD, the ability of Kupffer cells to release inflammatory factors increased greatly. In addition, NAFLD patients are often accompanied by disorders of adipokine levels, such as elevated CPR, IL-6, leptin, and reduced adiponectin levels, which make the body more prone to SIRS response [29]. Moreover there are reports that, in fatty liver mice and human, the reduction of alpha-1-antitrypsin (AAT) levels and the reduction of AAT can lead to excessive activation of inflammation [30].

Our study had the following limitations: it was retrospective, and the study population was not large enough. Second, it was a single-center study, and therefore, further research and verification are required in the future. And prospective studies are needed to demonstrate that NAFLD is a risk factor for a more severe pancreatitis. Third, liver biopsy is the gold standard for the diagnosis of NAFLD and other chronic liver diseases [31]. Previous studies showed that magnetic resonance imaging-based diagnostic methods are valuable in detecting NAFLD or determining the severity of NAFLD [32, 33]. However, in our study, an abdominal CT scan, which is our routine examination method, was used to diagnose NAFLD. This may have caused some data bias. Finally, the proportion of fatty pancreas in patients with NAFLD is higher than that in normal patients [34], and fatty pancreas may have an impact on AP. However, our study was a retrospective research, so we cannot be access to the data of fatty pancreas. Moreover, because of the fat hydrolysis in pancreatic tissue after AP onset, it is difficult to assess fat content in pancreas tissue in patients with AP.

## 5. Conclusions

In summary, our results demonstrated that the presence of NAFLD at admission portends a higher risk of moderately severe and SAP, as well as a higher risk of SIRS and organ failure. In the clinical environment, we should pay close attention to the phenomenon of NAFLD aggravation of the severity of AP.

## Figures and Tables

**Table 1:** Comparison of baseline and clinical characteristics between AP patients with versus without NAFLD.

Variables	Cohort	non-NAFLD	NAFLD	P
	N=656	n=278	n=378	
General situation				

Age (years) (mean ± SD)	43.93 ± 9.81	45.96 ± 10.20	42.44 ± 9.24	≤ 0.078

Male (%)	413 (63.0%)	157 (61.3%)	256 (67.7%)	0.004

BMI	25.99 ± 3.61	24.71 ± 3.43	26.90 ± 3.45	≤ 0.001

Smoking	134 (20.4%)	52 (18.7%)	82 (21.7%)	0.378

Etiology				≤ 0.001

Gallstone	207 (31.6%)	129 (49.0%)	78 (20.6%)	

Hypertriglyceridemia	316 (48.1%)	69 (26.2%)	247 (65.3%)	

Others	133 (20.3%)	80 (28.8%)	53 (14.1%)	

Basic disease				

Hypertension	51 (7.8%)	22 (7.9%)	29 (7.7%)	1.000

Diabetes	104 (15.9%)	32 (11.5%)	72 (19.0%)	0.009

CHD	9 (1.4%)	6 (2.1%)	3 (0.8%)	0.179

Laboratory indicators				

WBC (10^∧^9/L)	11.85 ± 4.97	11.17 ± 5.05	12.47 ± 4.89	0.001

ALT (IU/L)	40.00 (3, 1156)	40.45 (3, 1156)	39.50 (3, 793)	0.007

AST (IU/L)	35.00(8,850)	40.00 (8,850)	33.00 (8,850)	0.002

Cr (mmol/L)	59.00(30,1082)	57.00 (30,430)	60.00 (32,1082)	0.35

TG (mmol/L)	3.85(0.21,39.21)	1.48(0.21,30.48)	6.41 (0.24,39.21)	≤ 0.001

**Table 2:** Comparison of the Atlanta classification, BISAP score, Ranson score, SIRS, and organ failure with versus without NAFLD.

Variables	Cohort	non-NAFLD	NAFLD	P
	N=656	n=278	n=378	
Atlanta classification				≤ 0.001

MAP	435 (66.3%)	215 (77.3%)	220 (58.2%)	

MSAP	179 (27.3%)	51(18.3%)	128 (33.9%)	

SAP	42 (6.4%)	12 (4.3%)	30 (7.9%)	

BISAP Scores				0.006

<2	545 (83.1%)	245 (88.71%)	300 (79.3%)	

>=2	111 (16.9%)	33 (12.2%)	78 (20.7%)	

Ranson Scores				0.040

<3	576 (87.8%)	253 (91.0%)	323 (85.4%)	

>=3	80 (12.2%)	25 (9.0%)	55 (14.6%)	

SIRS				≤ 0.001

NO	432 (65.9%)	214 (77.0%)	218 (57.7%)	

YES	224 (34.1%)	64 (23.0%)	160 (42.3%)	

Organ Failure				≤ 0.001

NO	468 (66.9%)	215 (77.3%)	220 (58.2%)	

YES	232 (33.1%)	63 (22.7%)	158 (41.8%)	

**Table 3:** Comparison of the Atlanta classification and Ranson score, with non-NAFLD, mild-NAFL, and M+S-NAFL.

Variables	non-NAFLD	Mild-NAFLD	M+S-NAFLD	P
	n=278	n=234	n=144	
Atlanta classification				≤ 0.001

MAP	215 (77.4%)	149 (63.7%)	71 (49.3%)	

MSAP	51 (18.3%)	75 (32.1%)	53 (36.8%)	

SAP	12 (4.3%)	10 (4.3%)	20 (13.2%)	

Ranson Scores				0.003

<3	253 (91.0%)	208 (88.9%)	115 (79.9%)	

>=3	25 (9.0%)	26 (11.1%)	29 (20.1%)	

**Table 4:** Logistic regression analysis of organ failure in patients with AP.

	B	P	OR	95%CI
Non-NAFLD				
Mild-NAFLD	0.572	0.023	1.771	1.080-2.903
M+S-NAFLD	1.136	≤ 0.001	3.115	1.766-5.493
Male	0.053	0.824	1.055	0.660-1.685
Age	0.009	0.465	1.009	0.986-1.032
BMI	-0.006	0.843	0.994	0.934-1.057
TG	0.026	0.040	1.026	1.001-1.052
CHD	-0.930	0.397	0.395	0.046-3.401
Diabetes	0.388	0.142	1.474	0.878-2.474
Hypertension	0.248	0.555	1.282	0.562-2.927
Smoking	0.132	0.606	1.141	0.691-1.882

## Data Availability

All data generated and analyzed during this study are included in this published article. The datasets are available from the corresponding author on reasonable request.

## Abbreviations

AP: Acute pancreatitis
NAFLD: Nonalcoholic fatty liver disease
SIRS: Systemic inflammatory response syndrome
CT: Abdominal computed tomography
MAP: Mild AP
MSAP: Moderately severe AP
SAP: Severe AP
BMI: Body Mass Index
WBC: White blood cell
CI: Confidence interval
TG: Triglyceride concentrations
Cr: Creatinine
ALT: Alanine: transpeptidase
AST: Aspartate transaminase
HTG-AP: Hyperlipidemia pancreatitis
AAT: Alpha-1-antitrypsin
CHD: Coronary heart disease
M+S-NAFLD: Moderate-severe NAFLD.
